# Peptidylarginine Deiminase Inhibitor Suppresses Neutrophil Extracellular Trap Formation and MPO-ANCA Production

**DOI:** 10.3389/fimmu.2016.00227

**Published:** 2016-06-08

**Authors:** Yoshihiro Kusunoki, Daigo Nakazawa, Haruki Shida, Fumihiko Hattanda, Arina Miyoshi, Sakiko Masuda, Saori Nishio, Utano Tomaru, Tatsuya Atsumi, Akihiro Ishizu

**Affiliations:** ^1^Division of Rheumatology, Endocrinology and Nephrology, Hokkaido University Graduate School of Medicine, Sapporo, Japan; ^2^Faculty of Health Sciences, Hokkaido University, Sapporo, Japan; ^3^Department of Pathology, Hokkaido University Graduate School of Medicine, Sapporo, Japan

**Keywords:** MPO-ANCA-associated vasculitis, MPO-ANCA, neutrophil extracellular trap, peptidylarginine deiminase 4, peptidylarginine deiminase inhibitor

## Abstract

Myeloperoxidase-antineutrophil cytoplasmic antibody (MPO-ANCA)-associated vasculitis is a systemic small-vessel vasculitis, wherein, MPO-ANCA plays a critical role in the pathogenesis. Neutrophil extracellular traps (NETs) released from activated neutrophils are composed of extracellular web-like DNA and antimicrobial proteins, including MPO. Diverse stimuli, such as phorbol myristate acetate (PMA) and ligands of toll-like receptors (TLR), induce NETs. Although TLR-mediated NET formation can occur with preservation of living neutrophilic functions (called vital NETosis), PMA-stimulated neutrophils undergo cell death with NET formation (called suicidal NETosis). In the process of suicidal NETosis, histones are citrullinated by peptidylarginine deiminase 4 (PAD4). Since this step is necessary for decondensation of DNA, PAD4 plays a pivotal role in suicidal NETosis. Although NETs are essential for elimination of microorganisms, excessive formation of NETs has been suggested to be implicated in MPO-ANCA production. This study aimed to determine if pan-PAD inhibitors could suppress MPO-ANCA production *in vivo*. At first, NETs were induced in peripheral blood neutrophils derived from healthy donors (1 × 10^6^/ml) by stimulation with 20 nM PMA with or without 20 μM propylthiouracil (PTU), an anti-thyroid drug. We then determined that the *in vitro* NET formation was inhibited completely by 200 μM Cl-amidine, a pan-PAD inhibitor. Next, we established mouse models with MPO-ANCA production. BALB/c mice were given intraperitoneal (i.p.) injection of PMA (50 ng at days 0 and 7) and oral PTU (2.5 mg/day) for 2 weeks. These mice were divided into two groups; the first group was given daily i.p. injection of PBS (200 μl/day) (*n* = 13) and the other group with daily i.p. injection of Cl-amidine (0.3 mg/200 μl PBS/day) (*n* = 7). Two weeks later, citrullination as an indicator of NET formation in the peritoneum and serum MPO-ANCA titer was compared between the two groups. Results demonstrated that citrullination in the peritoneum was significantly reduced in the Cl-amidine-treated mice compared with the vehicle-injected control mice (38% reduction). Additionally, the serum MPO-ANCA titer of the Cl-amidine-treated mice (32.3 ± 31.0 ng/ml) was significantly lower than that in the vehicle-injected mice (132.1 ± 41.6 ng/ml). The collective findings indicate that excessive formation of NETs may be implicated in MPO-ANCA production *in vivo*.

## Introduction

Antineutrophil cytoplasmic antibody (ANCA)-associated vasculitis is a systemic small-vessel vasculitis (1). The major target antigens of ANCA are myeloperoxidase (MPO) and proteinase 3 (PR3). Neutrophils primed by pro-inflammatory cytokines, such as TNF-α, express MPO and PR3 on the cell surface. ANCA bind to the antigens and then activate neutrophils directly and/or through binding to bystander Fcγ receptors. Consequently, the activated neutrophils induce vascular endothelial cell injury resulting in the development of small-vessel vasculitis (2, 3). ANCA, therefore, play a critical role in the pathogenesis of ANCA-associated vasculitis. Although the mechanism of MPO-ANCA production was unknown for a long time, recent studies have suggested the involvement of neutrophil extracellular traps (NETs) in the mechanism (4–6).

Neutrophil extracellular traps are firstly reported in 2004 as extracellular web-like DNA studded with antimicrobial proteins, including MPO, which are released from phorbol myristate acetate (PMA)-stimulated neutrophils (7). The PMA-stimulated neutrophils undergo cell death with the formation of NETs (8), though not all stimuli induce cell death in NET-forming neutrophil (9). It has been demonstrated that NET formation can occur with preservation of living neutrophilic functions, including phagocytosis and chemotaxis (10, 11). Currently, NET formation undergoing cell death is called suicidal NETosis, whereas that preserves living neutrophilic functions is called vital NETosis. NETs can trap microorganisms by the extracellular DNA and kill them using the antimicrobial proteins. Thus, NETosis is considered as an important event in innate immunity. However, excessive NETosis can result in vascular endothelial cell injury (12), thrombosis (13, 14), and impairment of diabetic wound healing (15, 16). In addition, disordered regulation of NETosis has been suggested to be involved in the pathogenesis of autoimmune diseases, including systemic lupus erythematosus (SLE) (17) and anti-thyroid drug propylthiouracil (PTU)-induced MPO-ANCA-associated vasculitis (5). In the last two studies, the possibility of extracellular components in persistent NETs recognized as autoantigens by the immune system was discussed.

Suicidal NETosis is induced in response to diverse stimuli, including PMA (7, 18). These stimuli activate the Raf–mitogen-activated protein kinase kinase–extracellular signal-regulated kinase pathway, NADPH oxidase-dependent production of reactive oxygen species, and receptor-interacting protein kinase/mixed lineage kinase domain-like-mediated signals (19, 20). In this pathway, peptidylarginine deiminase 4 (PAD4) yields citrullination of histones, around which DNA coils.

The PAD enzymes convert arginine residues to citrulline in a variety of protein substrates (21). Among the PAD family, which includes PAD 1–4 and 6, PAD4 is expressed mainly in hematopoietic cells, such as neutrophils (22). In the process of suicidal NETosis, PAD4-dependent citrullination of histones that yields decondensation of DNA is an essential step to mix DNA and intracytoplasmic proteins. This mixture is subsequently extruded from the ruptured plasma membrane. Accordingly, PAD4 plays a pivotal role in the process of suicidal NETosis (23).

In the present study, we aimed to determine if a pan-PAD inhibitor, Cl-amidine, could suppress MPO-ANCA production *in vivo*. For this purpose, we attempted to generate novel mouse models of MPO-ANCA-associated vasculitis according to our previous protocol utilized for establishment of a rat model of this disease (5). The mouse models would be more useful models that require lower doses of reagents than the rat model.

## Materials and Methods

### Human Neutrophil Isolation

Human peripheral blood neutrophils were obtained from healthy volunteers by density centrifugation using Polymorphprep (Axis-Shield, Dundee, Scotland) according to the manufacturer’s instructions.

### NET Induction *In Vitro*

The *in vitro* NET induction was conducted similarly to our earlier study (5). In brief, human peripheral blood neutrophils were re-suspended in RPMI 1640 medium supplemented with 5% fetal bovine serum and then seeded in wells of 4-well chamber slides (Thermo Fisher Scientific, Yokohama, Japan) (1 × 10^6^/ml). After incubation for 30 min at 37°C, the cells were exposed to 0 or 20 nM PMA (Sigma-Aldrich, St. Louis, MO, USA) with or without 20 μM PTU (Chugai Pharmaceutical, Tokyo, Japan) and incubated for another 2 h at 37°C.

### PAD Inhibitor Administration *In Vitro*

Fifteen minutes prior to the PMA/PTU administration, 200 μM Cl-amidine (Calbiochem, San Diego, CA, USA), a pan-PAD inhibitor, was added alternately into the wells. The concentration of Cl-amidine was adopted according to the previous report (24).

### Quantification of NETs *In Vitro*

After 2 h of incubation, the medium containing the reagents was removed, and the remaining cells were washed with PBS followed by fixation with 4% paraformaldehyde for 15 min. Thereafter, the specimens were made to react with 5 μg/ml of rabbit anti-human citrullinated histone 3 polyclonal antibody (Abcam, Cambridge, UK) for 60 min at room temperature. After removal of unbound antibody, the specimens were next allowed to react with 1:500 dilution of Alexa Fluor 594-conjugated goat anti-rabbit IgG antibody (Invitrogen, Tokyo, Japan) for 60 min at room temperature. After washing with PBS, the specimens were finally mounted with the 4′, 6-diamidino-2-phenylindole (DAPI)-containing solution (Sigma-Aldrich). NET formation was observed under a fluorescent microscope and was quantified by counting the citrullinated histone 3-positive cells per × 100 power field of view. Data from five random fields of view (×100) were subjected to the quantitative analysis.

### Establishment of Mouse Models with MPO-ANCA Production

BALB/c, New Zealand White (NZW), C57BL/6N (B6/N), C57BL/6J (B6/J), and DBA mice (14-week-old female) were purchased from Clea Japan (Tokyo, Japan). The mice were given intraperitoneal (i.p.) injection of PMA (50 ng at days 0 and 7) and oral administration of PTU dissolved in 5% glucose water for 4 weeks (*n* = 5/strain). Since preliminary experiments revealed that each mouse ingested at least 2.5 ml of the drug-containing glucose water, the concentration of PTU was set as 1 mg/ml in order to administer exactly 2.5 mg PTU per day. These mice were maintained under specific pathogen-free condition in accordance with the guidelines for the care and use of laboratory animals in Hokkaido University (Permission No. 12-0077).

### Laboratory Data

Mouse urine was collected during the last 24 h using metabolic cages. Blood samples were obtained at days 14 and 28. Hematuria was assessed by dipsticks (Siemens Healthcare, Tokyo, Japan). Biochemical examinations for blood nitrogen urea (BUN) and creatinine (Cr) were performed at Daiichi Kishimoto Clinical Laboratory (Sapporo, Japan). Serum titer of MPO-ANCA was determined by enzyme-linked immunosorbent assay (ELISA) at A-CLIP Institute (Chiba, Japan).

### Histological Evaluation

The lungs, kidneys, and peritoneal tissues were obtained at day 28 and then fixed in 10% formalin. The pulmonary and renal sections were subjected to hematoxylin and eosin staining. The sections of the peritoneal tissues were subjected to immunohistochemistry for citrullinated histone 3 as described previously (25, 26).

### PAD Inhibitor Administration *In Vivo*

BALB/c mice (14-week-old female) were given i.p. injection of PMA (50 ng at days 0 and 7) and oral PTU (2.5 mg/day, aforementioned protocol) for 2 weeks. These mice were divided into two groups. The first group was given daily i.p. injection of PBS (200 μl/day) (*n* = 13). The second group was given daily i.p. injection of Cl-amidine (0.3 mg/200 μl PBS/day) (*n* = 7). The dose of Cl-amidine was adopted according to the previous report (27). Each mouse was administered at least 10 mg/kg of Cl-amidine per day. Mouse urine was collected during the last 24 h using metabolic cages. Blood and tissue samples were obtained at day 14.

### Statistical Analysis

The *in vitro* data were presented as mean ± standard deviation (SD) values obtained from experiments repeated for at least five times. The *in vivo* data were also presented as mean ± SD values. Mann–Whitney *U*-tests were applied for statistical evaluation with StatPlus software. *P*-values of <0.05 were regarded as statistically significant.

## Results

### Effect of PAD Inhibitor on NET Induction *In Vitro*

Human peripheral blood neutrophils were exposed to 0 or 20 nM PMA with or without 20 μM PTU. Fifteen minutes prior to PMA/PTU exposure, the neutrophils were treated with or without 200 μM Cl-amidine. Stimulation without PMA did not induce NETs (data not shown). It has been also determined that 200 μM of Cl-amidine did not affect the viability of neutrophils *in vitro* (Figure S1 in Supplementary Material).

**Figure 1:**
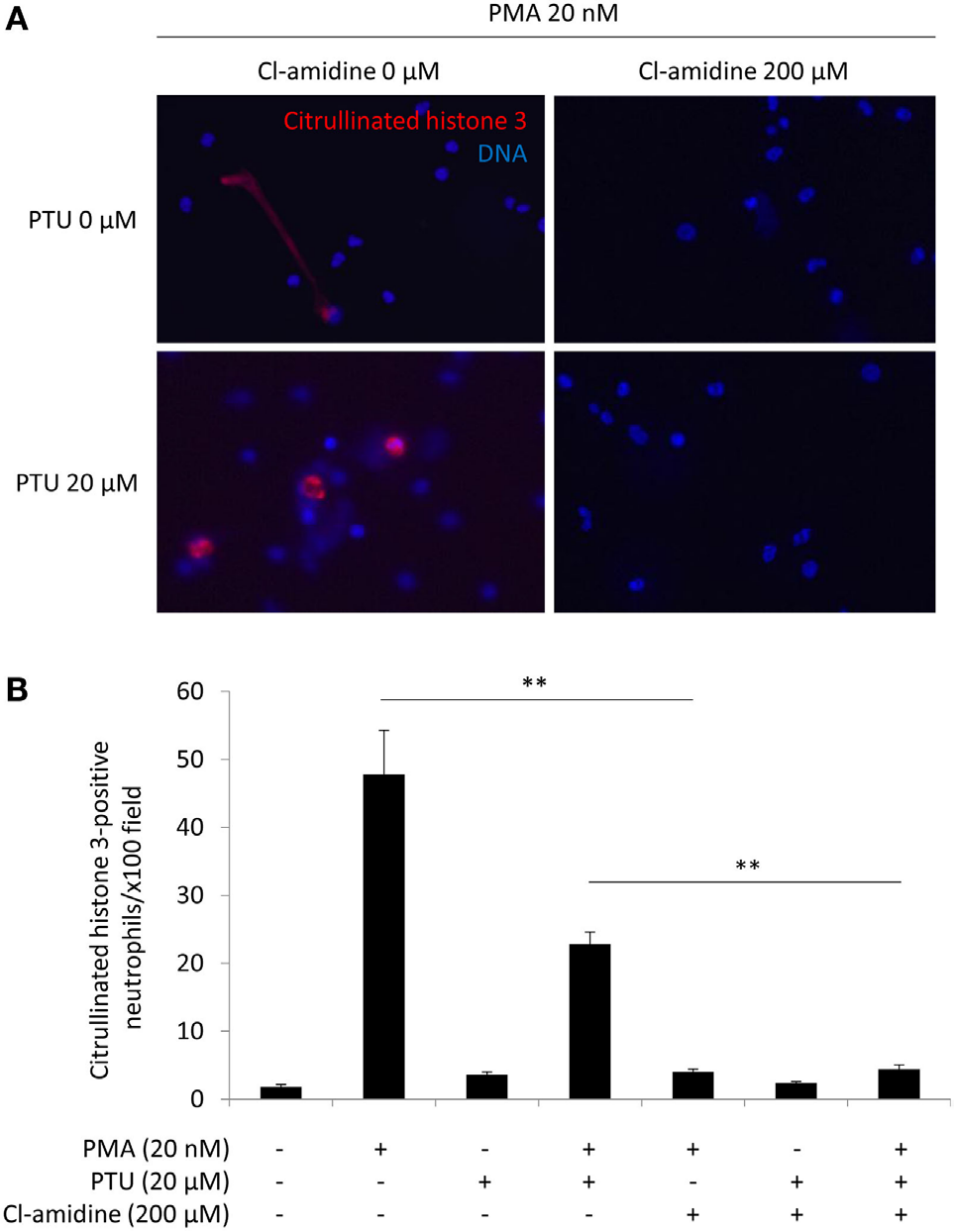
**Effect of Cl-amidine on NET induction** *in vitro*. Human peripheral blood neutrophils were seeded in wells of 4-well chamber slides (1 × 10^6^/ml). After incubation for 30 min at 37°C, the cells were exposed to 0 or 20 nM PMA with or without 20 μM PTU. Fifteen minutes prior to PMA/PTU administration, 200 μM Cl-amidine was added alternately into the wells. After 2 h of incubation at 37°C, the medium containing the reagents was removed, and the remaining cells were washed with PBS followed by fixation with 4% paraformaldehyde for 15 min. Thereafter, immunofluorescent staining for citrullinated histone 3 was carried out followed by mounting with the DAPI-containing solution. Representative photos **(A)**. Red, citrullinated histone 3; Blue: DNA (original magnification: ×400). Quantification of NET formation **(B)**. NET formation was quantified by counting the citrullinated histone 3-positive cells per × 100 power field of view. Data from five random fields of view (×100) were subjected to quantitative analysis. **p < 0.01 in Mann–Whitney *U*-test.

The neutrophils stimulated by 20 nM PMA alone formed extended NETs, whereas neutrophils stimulated by 20 nM PMA plus 20 μM PTU formed non-extended round-shaped NETs (Figure 1A). These findings corresponded with our earlier observations (5) and suggested that PTU yielded abnormal conformation of NETs induced by PMA. Although the morphology of NETs was different, both stimuli (PMA alone and PMA plus PTU) were shown to induce citrullination of histone 3 (5). Therefore, PAD4 was likely involved in the NET induction by these stimuli. As expected, the pan-PAD inhibitor, Cl-amidine (200 μM) inhibited the NET formation induced by both PMA alone and PMA plus PTU *in vitro* (Figures 1A,B). Furthermore, these findings suggest that Cl-amidine can inhibit not only the formation of PMA-induced conventional (extended) NETs but also PTU-mediated unconventional (non-extended round-shaped) NETs.

### Establishment of Mouse Models with MPO-ANCA Production

**Figure 2:**
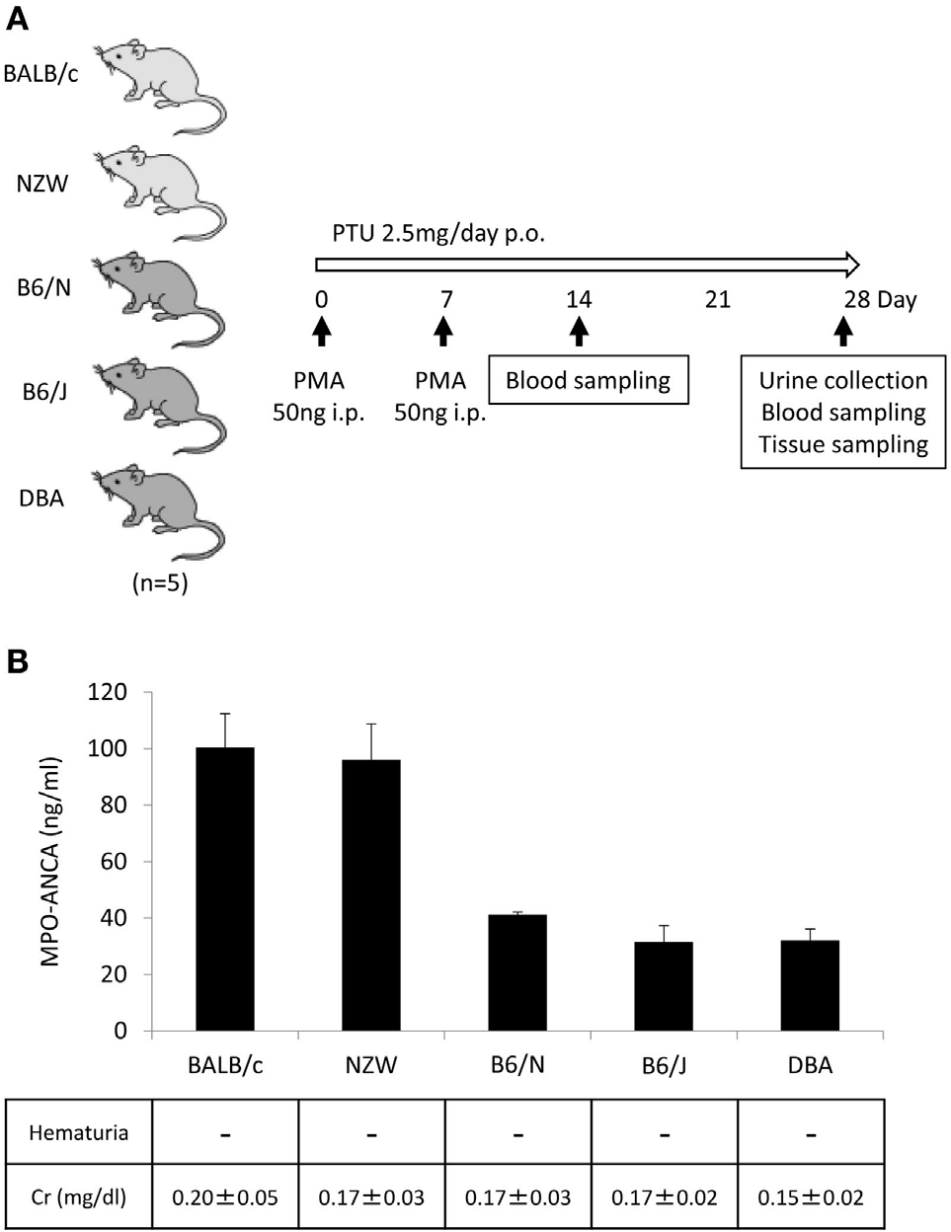
**Establishment of mouse models with MPO-ANCA production**. Experimental protocol **(A)**. BALB/c, NZW, B6/N, B6/J, and DBA mice (14-week-old female) were given i.p. injection of PMA (50 ng at days 0 and 7) and oral PTU (2.5 mg/day) for 4 weeks (*n* = 5/strain, p.o.: *Per Os*). Mouse urine was collected during the last 24 h using metabolic cages, and then hematuria was assessed by dipsticks. Blood samples were obtained at days 14 and 28, and then the concentrations of BUN and Cr were determined. The lungs, kidneys, and peritoneal tissues were obtained at day 28. Serum titers of MPO-ANCA determined by ELISA were shown with the results of the urinalysis and concentrations of Cr in the serum **(B)**. The normal limit of serum Cr level is 0.4 mg/dl.

In our earlier study, WKY rats were employed to establish an animal model of MPO-ANCA-associated vasculitis (5). In this study, we attempted to establish novel mouse models of MPO-ANCA-associated vasculitis according to the protocol for the rat model. For this purpose, BALB/c, NZW, B6/N, B6/J, and DBA mice (*n* = 5/strain) were given i.p. injection of PMA (50 ng at days 0 and 7) and oral PTU (2.5 mg/day) for 4 weeks (Figure 2A). The serum titers of MPO-ANCA at day 28 were 100.4 ± 12.0 ng/ml in BALB/c, 96.1 ± 12.8 ng/ml in NZW, 41.3 ± 0.90 ng/ml in B6/N, 31.6 ± 5.79 ng/ml in B6/J, and 32.0 ± 4.06 ng/ml in DBA mice (Figure 2B). Contrary to the rat model, no vasculitic lesion was observed in the lungs and kidneys of all mouse strains examined. Correspondingly, renal dysfunction was not detected in the urine and blood samples. Based on these findings, we employed BALB/c mice to construct the mouse model with MPO-ANCA production. The serum MPO-ANCA titer at day 14 was 79.0 ± 5.70 ng/ml in the BALB/c model. MPO-ANCA was not detected in the vehicle-injected BALB/c mice.

### Effect of PAD Inhibitor on Citrullination *In Vivo*

**Figure 3:**
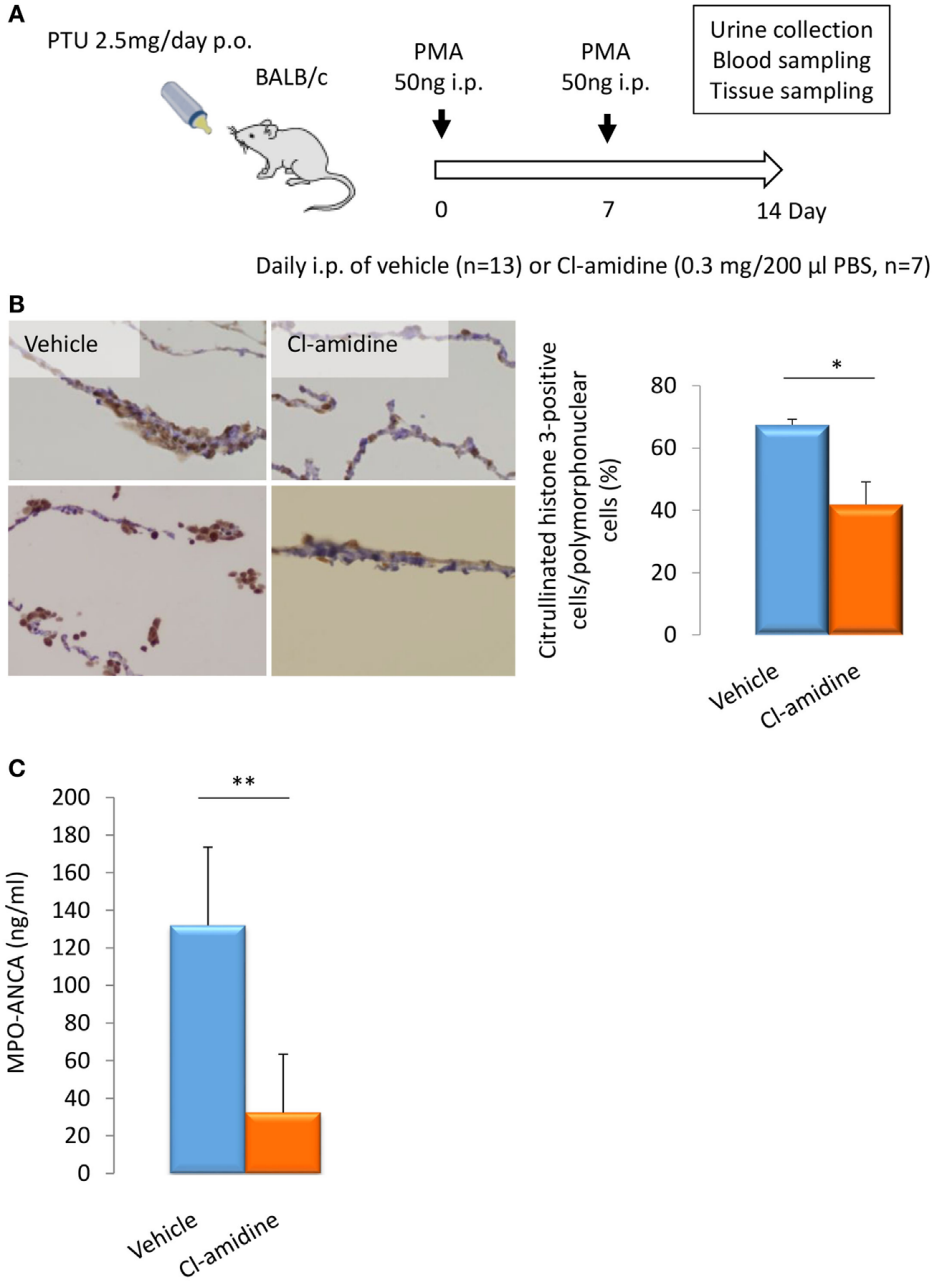
**Effect of Cl-amidine on citrullination and MPO-ANCA production *in vivo***. Experimental protocol **(A)**. BALB/c mice (14-week-old female) were given i.p. injection of PMA (50 ng at days 0 and 7) and oral PTU (2.5 mg/day) for 2 weeks (p.o.: *Per Os*). These mice were divided into two groups. The first group of mice was given daily i.p. injection of PBS (200 μl/day) (*n* = 13). The other group was given daily i.p. injection of Cl-amidine (0.3 mg/200 μl PBS/day) (*n* = 7). Mouse urine was collected during the last 24 h using metabolic cages. Blood and tissue samples were obtained at day 14. NET formation in peritoneal tissues **(B)**. The formalin-fixed paraffin-embedded sections of peritoneal tissues were subjected to immunohistochemistry for citrullinated histone 3. Representative photos among five random fields of view (×100) were shown. NET induction was quantified by calculating the rate of citrullinated histone 3-positive cells in polymorphonuclear cells in the five random fields of view. *p < 0.05 in Mann–Whitney *U*-test. Serum titers of MPO-ANCA determined by ELISA **(C)**. **p < 0.01 in Mann–Whitney *U*-test.

BALB/c mice (14-week-old female) were given i.p. injection of PMA (50 ng at days 0 and 7) and oral PTU (2.5 mg/day) for 2 weeks. These mice were divided into two groups. The first group was given daily i.p. injection of PBS (200 μl/day) (*n* = 13) and the other group with daily i.p. injection of Cl-amidine (0.3 mg/200 μl PBS/day) (*n* = 7) (Figure 3A). At day 14, the peritoneal tissues were subjected to immunohistochemistry for citrullinated histone 3 for the evaluation of NET formation *in vivo*. As a result, the rate of citrullinated histone 3-positive cells in polymorphonuclear cells was significantly reduced in the Cl-amidine-treated mice in comparison with the vehicle-injected control mice (38% reduction) (Figure 3B).

### Effect of PAD Inhibitor on MPO-ANCA Production *In Vivo*

The serum titers of MPO-ANCA in the Cl-amidine-treated mice (32.3 ± 31.0 ng/ml) were significantly lower than the vehicle-injected control mice (132.1 ± 41.6 ng/ml) (Figure 3C). The collective findings clearly indicate that NET formation is inhibited by the pan-PAD inhibitor, Cl-amidine, both *in vitro* and *in vivo*, and that MPO-ANCA production is suppressed by Cl-amidine *in vivo*.

## Discussion

The PMA-stimulated neutrophils extrude decondensated DNA, which forms extracellular web-like structures decorated with bactericidal proteins (7). Since this substance, called NETs, can bind and kill bacteria, NET formation is regarded as an important event in innate immunity. Chronic granulomatous disease (CGD) patients who cannot generate NETs are susceptible to diverse bacteria and fungi indeed, and it was shown that restoration of NET formation in CGD resulted in resistance to such infections (28). Currently, it is considered that NET appears to be a form of innate response that binds microorganisms, prevents them from spreading, and ensures a high local concentration of antimicrobial agents derived from neutrophils (29).

The PMA-stimulated neutrophils undergo cell death with NET formation (8). Since the characteristics of cell death resembled neither typical necrosis nor apoptosis, Steinberg et al. coined NETosis for the neutrophil death with NET formation (30). However, Clark et al. have demonstrated that neutrophils do not necessarily undergo cell death after NET formation (31). It has been shown that NET formation can occur with preservation of neutrophilic functions, including phagocytosis and chemotaxis (10, 11). This phenomenon termed vital NETosis (9). On the contrary, aforementioned dying NETosis has been called suicidal NETosis.

Although NETs play an essential role in the innate immunity, some harmful aspects of NETs have been demonstrated (32, 33). They not only have direct cytotoxic and thrombotic effects on vascular endothelium (12–14), but NETs are also associated with pathogenic autoantibody production (5, 17). For example, impairment of NET degradation potential (low activity of DNase I) in the serum is present in 30–40% of patients with SLE and is suggested to lead the production of anti-DNA antibodies, which are related to disease severity (17). In another case, morphologically abnormal and DNase I-resistant NETs are generated by the anti-thyroid drug, PTU, and then MPO in the NETs seems to be recognized by the immune system resulting in the production of MPO-ANCA in rats administered with PTU (5). It has been shown that a part of PTU is metabolized by MPO. Simultaneously, conformational alteration of MPO could be induced by PTU (34), which could result in the tolerance break to MPO.

In the present study, we established mouse models with MPO-ANCA production. We treated five strains of mouse with PMA and PTU corresponding to the previously established protocol for the rat model of MPO-ANCA-associated vasculitis. Interestingly, there was a strain-dependency in the response to produce MPO-ANCA in mouse. Although the precise mechanism of the strain-dependency has to be determined in future studies, the Th2 phenotype of BALB/c (35) and autoimmune-prone genetic background of NZW (36) may be associated with the susceptibility to produce autoantibodies. In addition, no vasculitic lesion was observed in the mouse models with MPO-ANCA production, whereas WKY rats with MPO-ANCA developed pulmonary capillaritis and glomerulonephritis (5). Species-dependency may be present in the development of PMA plus PTU-induced MPO-ANCA-associated vasculitis. Nonetheless, we employed BALB/c mice to construct *in vivo* models of MPO-ANCA production.

Neeli et al. first demonstrated that PAD4-dependent histone deimination occurred in activated neutrophils under inflammatory conditions (37). Since PAD4-deficient neutrophils did not generate NETs in response to PMA, PAD4 plays a pivotal role in the NET formation (23). Correspondingly, inhibition of PAD4 using the pan-PAD inhibitor, Cl-amidine, prevented citrullination of histone 3 and significantly reduced NET release from HL60 cells, which were differentiated into mature neutrophils, in response to Ca^2+^ ionophore or *Shigella flexneri* exposure (24). In addition, Cl-amidine has been shown to suppress NET formation in lupus-prone mice (27). It has been shown that Cl-amidine can modify the cysteine of PAD and then inactivate it irreversibly (38). Based on these findings, we conducted *in vitro* and *in vivo* experiments to inhibit citrullination/NET formation using Cl-amidine as a pan-PAD inhibitor. The present study reproduced and extended the results of previous reports that investigated Cl-amidine both *in vitro* and *in vivo* and demonstrated that MPO-ANCA production was suppressed by Cl-amidine *in vivo*. These findings suggest that excessive formation of NETs may be implicated in MPO-ANCA production *in vivo*. In order to demonstrate the direct implication of PAD4-dependent NET formation in MPO-ANCA production, further studies using PAD4-deficient mice should be designed.

One limitation of this study is the lack of quantification of released NETs, which can be usually detected as MPO-DNA complexes in the serum. However, it has been shown that the PMA plus PTU-induced NETs hardly converted into soluble form (5). Thus, there is no better methodology to evaluate NETs in the murine model than the immunohistochemistry for citrullinated histone 3.

No vasculitic phenotype was observed in the PMA plus PTU-induced mouse models with MPO-ANCA production. Therefore, we could not examine the effect of PAD inhibitors on MPO-ANCA-associated vasculitis in this model, which is another limitation of this study. It should be determined whether increased doses of PMA/PTU and/or longer duration could induce vasculitis in the mouse models in future studies.

It has been shown that MPO-ANCA is the major pathogenic factor in MPO-ANCA-associated vasculitis (2, 3). Thus, it is expected that PAD inhibitors, which can suppress the production of the pathogenic autoantibody, would be applied for the treatment of patients with MPO-ANCA-associated vasculitis. Preceding studies have demonstrated the protective effects of PAD inhibitors on the models of SLE (27) and other NET-related diseases, including multiple sclerosis (39), collagen-induced arthritis (40), and inflammatory bowel disease (41). Although further studies are needed to clarify their safety and effectiveness, PAD inhibitors are potential candidates as novel therapeutic agents for various NET-related diseases, including MPO-ANCA-associated vasculitis.

## Author Contributions

YK, HS, FH, and AM performed the experiments. YK, DN, SM, SN, UT, TA, and AI analyzed and discussed the data. YK, DN, UT, and AI designed the research. YK, UT, and AI wrote the manuscript.

## Conflict of Interest Statement

The authors declare that the research was conducted in the absence of any commercial or financial relationships that could be construed as a potential conflict of interest.

## References

[1] Jennette JC Falk RJ Bacon PA Basu N Cid MC Ferrario F 2012 revised international chapel hill consensus conference nomenclature of vasculitides. Arthritis Rheum (2013) 65:1–11. 10.1002/art.37715 23045170

[2] Jennette JC Falk RJ. Pathogenesis of antineutrophil cytoplasmic autoantibody-mediated disease. Nat Rev Rheumatol (2014) 10:463–73. 10.1038/nrrheum.2014.103 25003769

[3] Kallenberg CG Pathogenesis and treatment of ANCA-associated vasculitides. Clin Exp Rheumatol (2015) 33:S11–4. 26457917

[4] Kessenbrock K Krumbholz M Schonermarck U Back W Gross WL Werb Z Netting neutrophils in autoimmune small-vessel vasculitis. Nat Med (2009) 15:623–5. 10.1038/nm.1959 19448636 PMC2760083

[5] Nakazawa D Tomaru U Suzuki A Masuda S Hasegawa R Kobayashi T Abnormal conformation and impaired degradation of propylthiouracil-induced neutrophil extracellular traps: implications of disordered neutrophil extracellular traps in a rat model of myeloperoxidase antineutrophil cytoplasmic antibody-associated vasculitis. Arthritis Rheum (2012) 64:3779–87. 10.1002/art.34619 22777766

[6] Sangaletti S Tripodo C Chiodoni C Guarnotta C Cappetti B Casalini P Neutrophil extracellular traps mediate transfer of cytoplasmic neutrophil antigens to myeloid dendritic cells toward ANCA induction and associated autoimmunity. Blood (2012) 120:3007–18. 10.1182/blood-2012-03-416156 22932797

[7] Brinkmann V Reichard U Goosmann C Fauler B Uhlemann Y Weiss DS Neutrophil extracellular traps kill bacteria. Science (2004) 303:1532–5. 10.1126/science.1092385 15001782

[8] Fuchs TA Abed U Goosmann C Hurwitz R Schulze I Wahn V Novel cell death program leads to neutrophil extracellular traps. J Cell Biol (2007) 176:231–41. 10.1083/jcb.200606027 17210947 PMC2063942

[9] Yipp BG Kubes P. NETosis: how vital is it? Blood (2013) 122:2784–94. 10.1182/blood-2013-04-457671 24009232

[10] Pilsczek FH Salina D Poon KK Fahey C Yipp BG Sibley CD A novel mechanism of rapid nuclear neutrophil extracellular trap formation in response to *Staphylococcus aureus*. J Immunol (2010) 185:7413–25. 10.4049/jimmunol.1000675 21098229

[11] Yipp BG Petri B Salina D Jenne CN Scott BN Zbytnuik LD Infection-induced NETosis is a dynamic process involving neutrophil multitasking in vivo. Nat Med (2012) 18:1386–93. 10.1038/nm.2847 22922410 PMC4529131

[12] Xu J Zhang X Pelayo R Monestier M Ammollo CT Semeraro F Extracellular histones are major mediators of death in sepsis. Nat Med (2009) 15:1318–21. 10.1038/nm.2053 19855397 PMC2783754

[13] Fuchs TA Brill A Duerschmied D Schatzberg D Monestier M Myers DD Jr Extracellular DNA traps promote thrombosis. Proc Natl Acad Sci U S A (2010) 107:15880–5. 10.1073/pnas.1005743107 20798043 PMC2936604

[14] Fuchs TA Brill A Wagner DD. Neutrophil extracellular trap (NET) impact on deep vein thrombosis. Arterioscler Thromb Vasc Biol (2012) 32:1777–83. 10.1161/ATVBAHA.111.242859 22652600 PMC3495595

[15] Wong SL Demers M Martinod K Gallant M Wang Y Goldfine AB Diabetes primes neutrophils to undergo NETosis, which impairs wound healing. Nat Med (2015) 21:815–9. 10.1038/nm.3887 26076037 PMC4631120

[16] Fadini GP Menegazzo L Rigato M Scattolini V Poncina N Bruttocao A NETosis delays diabetic wound healing in mice and humans. Diabetes (2016) 65(4):1061–71. 10.2337/db15-0863 26740598

[17] Hakkim A Furnrohr BG Amann K Laube B Abed UA Brinkmann V Impairment of neutrophil extracellular trap degradation is associated with lupus nephritis. Proc Natl Acad Sci U S A (2010) 107:9813–8. 10.1073/pnas.0909927107 20439745 PMC2906830

[18] Yan J Meng X Wancket LM Lintner K Nelin LD Chen B Glutathione reductase facilitates host defense by sustaining phagocytic oxidative burst and promoting the development of neutrophil extracellular traps. J Immunol (2012) 188:2316–27. 10.4049/jimmunol.1102683 22279102 PMC3480216

[19] Hakkim A Fuchs TA Martinez NE Hess S Prinz H Zychlinsky A Activation of the Raf-MEK-ERK pathway is required for neutrophil extracellular trap formation. Nat Chem Biol (2011) 7:75–7. 10.1038/nchembio.496 21170021

[20] Desai J Kumar SV Mulay SR Konrad L Romoli S Schauer C PMA and crystal-induced neutrophil extracellular trap formation involves RIPK1-RIPK3-MLKL signaling. Eur J Immunol (2016) 46:223–9. 10.1002/eji.201545605 26531064

[21] Rohrbach AS Slade DJ Thompson PR Mowen KA. Activation of PAD4 in NET formation. Front Immunol (2012) 3:360. 10.3389/fimmu.2012.00360 23264775 PMC3525017

[22] Wang S Wang Y. Peptidylarginine deiminases in citrullination, gene regulation, health and pathogenesis. Biochim Biophys Acta (2013) 1829:1126–35. 10.1016/j.bbagrm.2013.07.003 23860259 PMC3775966

[23] Li P Li M Lindberg MR Kennett MJ Xiong N Wang Y. PAD4 is essential for antibacterial innate immunity mediated by neutrophil extracellular traps. J Exp Med (2010) 207:1853–62. 10.1084/jem.20100239 20733033 PMC2931169

[24] Wang Y Li M Stadler S Correll S Li P Wang D Histone hypercitrullination mediates chromatin decondensation and neutrophil extracellular trap formation. J Cell Biol (2009) 184:205–13. 10.1083/jcb.200806072 19153223 PMC2654299

[25] Nakazawa D Tomaru U Yamamoto C Jodo S Ishizu A Abundant neutrophil extracellular traps in thrombus of patient with microscopic polyangiitis. Front Immunol (2012) 3:333 10.3389/fimmu.2012.00333 23162551 PMC3495275

[26] Imamoto T Nakazawa D Shida H Suzuki A Otsuka N Tomaru U Possible linkage between microscopic polyangiitis and thrombosis via neutrophil extracellular traps. Clin Exp Rheumatol (2014) 32:149–50. 24321560

[27] Knight JS Zhao W Luo W Subramanian V O’dell AA Yalavarthi S Peptidylarginine deiminase inhibition is immunomodulatory and vasculoprotective in murine lupus. J Clin Invest (2013) 123:2981–93. 10.1172/JCI67390 23722903 PMC3696545

[28] Bianchi M Hakkim A Brinkmann V Siler U Seger RA Zychlinsky A Restoration of NET formation by gene therapy in CGD controls aspergillosis. Blood (2009) 114:2619–22. 10.1182/blood-2009-05-221606 19541821 PMC2756123

[29] Papayannopoulos V Zychlinsky A. NETs: a new strategy for using old weapons. Trends Immunol (2009) 30:513–21. 10.1016/j.it.2009.07.011 19699684

[30] Steinberg BE Grinstein S. Unconventional roles of the NADPH oxidase: signaling, ion homeostasis, and cell death. Sci STKE (2007) 2007:e11. 10.1126/stke.3792007pe11 17392241

[31] Clark SR Ma AC Tavener SA McDonald B Goodarzi Z Kelly MM Platelet TLR4 activates neutrophil extracellular traps to ensnare bacteria in septic blood. Nat Med (2007) 13:463–9. 10.1038/nm1565 17384648

[32] Doring Y Weber C Soehnlein O Footprints of neutrophil extracellular traps as predictors of cardiovascular risk. Arterioscler Thromb Vasc Biol (2013) 33:1735–6. 10.1161/ATVBAHA.113.301889 23818484

[33] Grayson PC Kaplan MJ. At the bench: neutrophil extracellular traps (NETs) highlight novel aspects of innate immune system involvement in autoimmune diseases. J Leukoc Biol (2016) 99:253–64. 10.1189/jlb.5BT0615-247R 26432901 PMC4718195

[34] Lee E Hirouchi M Hosokawa M Sayo H Kohno M Kariya K. Inactivation of peroxidases of rat bone marrow by repeated administration of propylthiouracil is accompanied by a change in the heme structure. Biochem Pharmacol (1988) 37:2151–3. 10.1016/0006-2952(88)90574-6 2837228

[35] Huang XR Holdsworth SR Tipping PG. Th2 responses induce humorally mediated injury in experimental anti-glomerular basement membrane glomerulonephritis. J Am Soc Nephrol (1997) 8:1101–8. 9219159 10.1681/ASN.V871101

[36] Lang J Bellgrau D. A T-cell functional phenotype common among autoimmune-prone rodent strains. Scand J Immunol (2002) 55:546–59. 10.1046/j.1365-3083.2002.01086.x 12028557

[37] Neeli I Khan SN Radic M. Histone deimination as a response to inflammatory stimuli in neutrophils. J Immunol (2008) 180:1895–902. 10.4049/jimmunol.180.3.1895 18209087

[38] Luo Y Arita K Bhatia M Knuckley B Lee YH Stallcup MR Inhibitors and inactivators of protein arginine deiminase 4: functional and structural characterization. Biochemistry (2006) 45:11727–36. 10.1021/bi061180d 17002273 PMC1808342

[39] Moscarello MA Lei H Mastronardi FG Winer S Tsui H Li Z Inhibition of peptidyl-arginine deiminases reverses protein-hypercitrullination and disease in mouse models of multiple sclerosis. Dis Model Mech (2013) 6:467–78. 10.1242/dmm.010520 23118341 PMC3597028

[40] Willis VC Gizinski AM Banda NK Causey CP Knuckley B Cordova KN N-alpha-benzoyl-N5-(2-chloro-1-iminoethyl)-L-ornithine amide, a protein arginine deiminase inhibitor, reduces the severity of murine collagen-induced arthritis. J Immunol (2011) 186:4396–404. 10.4049/jimmunol.1001620 21346230 PMC3085980

[41] Chumanevich AA Causey CP Knuckley BA Jones JE Poudyal D Chumanevich AP Suppression of colitis in mice by Cl-amidine: a novel peptidylarginine deiminase inhibitor. Am J Physiol Gastrointest Liver Physiol (2011) 300:G929–38. 10.1152/ajpgi.00435.2010 21415415 PMC3119113

