# Supplementary material for: Peptidylarginine Deiminase Inhibitor Suppresses Neutrophil Extracellular Trap Formation and MPO-ANCA Production
Source: Front Immunol. 2016 Jun 8;7:227. doi: 10.3389/fimmu.2016.00227 (PMC4896908; doi:10.3389/fimmu.2016.00227)
Supplement: Supplementary file 1 [file image_1.pdf]

No stimulation | Cl-amidine 200 μM

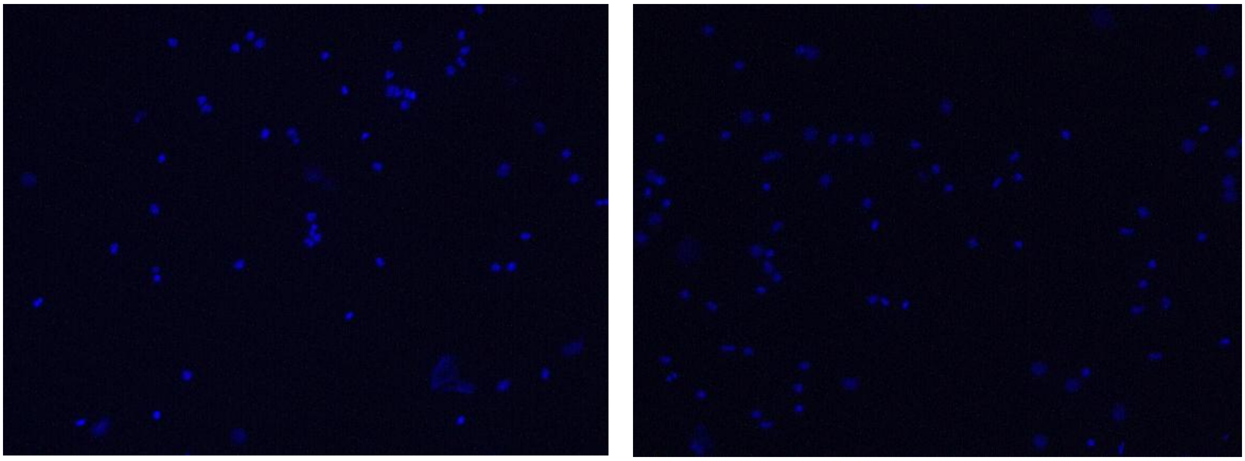

FIGURE S1. Effect of Cl-amidine on neutrophils *in vitro.*

Human peripheral blood neutrophils were seeded in wells of 4-well chamber slides (1 × 10^6/ml). After incubation for 30 minutes at 37 ℃, the cells were exposed to 0 or 200 μM Cl-amidine. After 2 hours of incubation at 37 ℃, the medium was removed, and the remaining cells were washed with PBS followed by fixation with 4% paraformaldehyde for 15 minutes. Thereafter, the specimens were made to react with 5 μg/ml of rabbit anti-human citrullinated histone 3 polyclonal antibody for 60 minutes at room temperature. After removal of unbound antibody, the specimens were next allowed to react with 1:500 dilution of Alexa Fluor 594-conjugated goat anti-rabbit IgG antibody for 60minutes at room temperature. After washing with PBS, the specimens were finally mounted with the DAPI-containing solution. Representative photos were shown (original magnification: ×100). There was no red signal that represented the formation of NETs. The nuclear morphology seen in blue suggested no cytotoxic effect of Cl-amidine (200 μM) on neutrophils.
